# Redox Imbalance in the Cardiohepatic Syndrome: The Emerging Role of Oxidative Stress in Cirrhosis-Associated Cardiac Dysfunction

**DOI:** 10.3390/antiox15040490

**Published:** 2026-04-15

**Authors:** Nikola Blagojevic, Dragana Blagojevic, Ana Matovic, Marko Cvrkotic, Marija Marjanovic-Haljilji, Aleksandra Sljivic, Ana Ilic, Natasa Cvetinovic, Irina Nenadic, Marko Djuric, Nemanja Dimic, Milica Aleksic, Jovana Bojicic, Aleksandra Djokovic, Snezana Lukic, Branka Filipovic

**Affiliations:** 1 Clinic for Internal Medicine, Cardiology Department, University Clinical Hospital Center “Dr Dragisa Misovic–Dedinje”, 11000 Belgrade, Serbia; 2 Clinic for Internal Medicine, Gastroenterology and Hepatology Department, University Clinical Hospital Center “Dr Dragisa Misovic–Dedinje”, 11000 Belgrade, Serbia; 3 Institute for Cardiovascular Diseases “Dedinje”, 11000 Belgrade, Serbia; 4 Faculty of Medicine, University of Belgrade, 11000 Belgrade, Serbia; 5 Clinic for Anesthesiology and Intensive Care, University Clinical Hospital Center “Dr Dragisa Misovic–Dedinje”, 11000 Belgrade, Serbia; 6 Center for Physical Medicine and Rehabilitation, University Clinical Center of Serbia, 11000 Belgrade, Serbia; 7 Clinic for Surgery, University Clinical Hospital Center “Zvezdara”, 11000 Belgrade, Serbia; 8 Clinic for Internal Medicine, Cardiology Department, University Clinical Hospital Center “Bezanijska Kosa”, 11000 Belgrade, Serbia; 9 Clinic for Gastroenterology and Hepatology, University Clinical Center of Serbia, 11000 Belgrade, Serbia

**Keywords:** cardiohepatic syndrome, oxidative stress, cirrhosis-associated cardiac dysfunction

## Abstract

Cirrhosis is no longer viewed solely as an isolated hepatic disorder but rather as a complex multisystemic disease that affects cardiovascular, renal, pulmonary, metabolic, and immune systems. One of its most clinically relevant but under-recognized consequences is cardiac dysfunction, manifesting as cirrhotic cardiomyopathy, portopulmonary hypertension, right ventricular (RV) failure, and impaired myocardial strain. Oxidative stress (OS) has recently emerged as a fundamental mechanistic link between hepatic fibrogenesis and myocardial remodeling, acting through mitochondrial injury, NADPH oxidase activation, nitric oxide dysregulation, iron-mediated ferroptosis, and inflammatory cytokines. These alterations lead to diastolic dysfunction, autonomic imbalance, myocardial fibrosis, electrophysiological abnormalities (including QTc prolongation), and impaired RV–pulmonary artery coupling. Redox biomarkers such as malondialdehyde (MDA), NOX2-derived peptides, GSH/GSSG ratio, sST2, NT-proBNP, and 8-isoprostanes hold promise in detecting early subclinical cardiac involvement in cirrhosis. Novel antioxidant therapies, including mitochondrial-targeted molecules, NOX inhibitors, and ferroptosis blockers, may improve myocardial remodeling and hemodynamic stability. This review explores the central role of redox imbalance in the cardiohepatic syndrome and its potential utility in diagnosis, monitoring, and therapy.

## 1. Introduction

Cirrhosis is a progressive liver disease characterized by chronic inflammation, hepatocellular injury, and extensive extracellular matrix deposition, leading to fibrosis, architectural distortion, and portal hypertension [1,2]. Historically considered a liver-restricted disorder, cirrhosis is now recognized as a systemic disease associated with substantial extrahepatic consequences, including immunologic dysfunction, renal impairment, pulmonary vasculopathy, endocrine disruption, sarcopenia, and cardiovascular involvement [3,4].

One of the most clinically significant but often underdiagnosed cardiac manifestations of liver disease is cirrhotic cardiomyopathy, characterized by blunted contractile response to stress, diastolic dysfunction, electrophysiological abnormalities (particularly QT prolongation), autonomic dysregulation, and subclinical systolic impairment [5,6,7]. Unlike classic heart failure, cirrhotic cardiomyopathy is usually silent at rest due to compensatory vasodilation and low afterload but becomes evident during physiologic stress such as infections, hemorrhage, transjugular intrahepatic portosystemic shunt (TIPS) insertion, or liver transplantation [8].

Alongside cirrhotic cardiomyopathy, other components of the cardiohepatic spectrum include portopulmonary hypertension (PoPH), hepatopulmonary syndrome (HPS), and congestive hepatopathy, reflecting the bidirectional pathophysiological links between the liver and heart [9,10]. While congestive hepatopathy results from chronic right-sided heart failure, leading to passive hepatic congestion and fibrosis, cirrhosis-induced cardiac dysfunction is mediated by hemodynamic stress, inflammatory activation, nitric oxide dysregulation, neurohormonal imbalance, and oxidative stress (OS) [11].

### 1.1. Oxidative Stress: An Emerging Pathogenic Bridge

Oxidative stress refers to an imbalance between the generation of reactive oxygen species (ROS) and reactive nitrogen species (RNS), and the ability of antioxidant defense systems—such as glutathione (GSH), superoxide dismutase (SOD), and catalase—to neutralize them [12]. In cirrhosis, excessive ROS originate from NADPH oxidase (NOX) activation, mitochondrial damage, CYP2E1 metabolism, xanthine oxidase activity, and iron-catalyzed Fenton reactions [13,14]. ROS-driven modifications affect not only hepatocytes but also cardiomyocytes, vascular endothelial cells, cardiac fibroblasts, and pulmonary vascular smooth muscle, contributing to myocardial fibrosis, ventricular stiffening, endothelial dysfunction, and RV failure [15].

Several features establish oxidative stress as a mechanistic link connecting hepatic fibrosis and cardiac dysfunction: (a) NADPH oxidase activation promotes profibrotic signaling in both hepatic stellate cells and cardiomyocytes [16,17]; (b) mitochondrial injury leads to ATP depletion, apoptosis, and structural remodeling in both hepatic and cardiac tissues [18,19]; (c) ferroptosis and iron overload accelerate hepatic fibrosis and cardiomyocyte death [20,21]; (d) nitrosative stress contributes to QT prolongation and impaired diastolic function [22,23]; (e) portal hypertension-induced endothelial dysfunction also contributes to pulmonary hypertension [24,25].

### 1.2. Hemodynamic, Neurohumoral, and Inflammatory Mediators

The hyperdynamic circulation characteristic of cirrhosis—marked by elevated cardiac output, reduced systemic vascular resistance, and increased plasma volume—is mediated by nitric oxide overproduction and splanchnic vasodilation [26]. Over time, RAAS activation, sympathetic activation, and endothelin release lead to myocardial hypertrophy and fibrosis [27]. Systemic inflammation and peripheral arterial vasodilation contribute to neurohormonal activation, circulatory dysfunction, and organ remodeling in cirrhosis [28].

Unlike classical heart failure, cirrhotic cardiomyopathy has preserved ejection fraction but impaired diastolic function and contractile reserve. Echocardiography reveals increased left atrial size, elevated E/e′ ratio, and RV dysfunction, especially under stress [29]. Emerging data indicate that pulmonary vascular abnormalities associated with cirrhosis—including nitric oxide-mediated vasodilation and impaired vascular regulation—may contribute to cardiopulmonary dysfunction in advanced liver disease [30].

### 1.3. Clinical Relevance and Unmet Needs

The clinical impact of cardiac dysfunction in cirrhosis has been underestimated. It plays a crucial role in prognosis, risk stratification for transplantation, and mortality during acute-on-chronic liver failure (ACLF) [31,32]. Oxidative stress-mediated cardiac injury correlates with inflammation, fibrosis stage, and survival [33]. Current clinical tools lack sensitivity for early detection, especially for RV dysfunction and subclinical myocardial strain impairment [34,35].

Taken together, these observations suggest a conceptual framework in which echocardiographic strain measurements, integrated with redox-sensitive biomarkers (MDA, NOX2, GSH/GSSG), might permit earlier detection of cirrhosis-related cardiac dysfunction. In this scenario, a unified cardiohepatic risk module could prove particularly informative for TIPS candidacy, transplant assessment, and PoPH surveillance.

## 2. The Cardiohepatic Axis: Pathophysiological Interactions Between the Liver and the Heart

**Figure 1:**
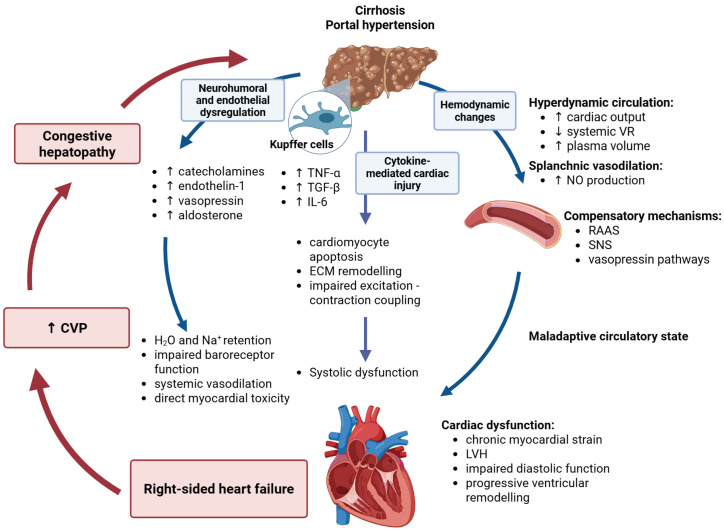
**The cardiohepatic axis–pathophysiology.** Liver cirrhosis and portal hypertension induce hyperdynamic circulation and splanchnic vasodilation, activating compensatory pathways including RAAS, the sympathetic nervous system, and vasopressin signaling. Hepatic inflammation, driven by activated Kupffer cells, promotes cytokine release (e.g., TNF-α, IL-6, TGF-β), leading to myocardial injury through effects on cardiomyocytes (apoptosis, impaired excitation–contraction coupling) and endothelial cells (dysfunction and altered vascular tone). These processes result in systolic and diastolic dysfunction and progressive cardiac remodeling. Neurohumoral activation further contributes to fluid retention and increased central venous pressure (CVP), promoting right-sided heart failure and hepatic congestion. In addition to these interactions, redox imbalance—driven by mitochondrial dysfunction and NADPH oxidase (NOX)-derived reactive oxygen species—amplifies inflammatory, endothelial, and myocardial injury pathways across the cardiohepatic axis. (VR—vascular resistance; NO—nitrite oxide; RAAS—renin–angiotensin–aldosterone system; SNS—sympathetic nervous system; TNF-α—tumor necrosis factor α; IL-6—interleukin 6; TGF-β—transcriptional growth factor β; ECM—extracellular matrix; LVH—left ventricular hypertrophy; CVP—central venous pressure). Created in BioRender. Blagojević, N. (2026) https://BioRender.com/zitj4b4, accessed on 6 April 2026.

The cardiohepatic axis represents the bidirectional relationship between liver disease and cardiac dysfunction. Cirrhosis leads not only to hepatic architectural distortion and portal hypertension but also to significant systemic effects impacting the cardiovascular system. Conversely, cardiac dysfunction—particularly right-sided heart failure—can induce hepatic congestion and fibrosis, creating a condition known as congestive hepatopathy [36] Figure 1.

### 2.1. Hemodynamic Changes and Hyperdynamic Circulation

A hallmark feature of liver cirrhosis is hyperdynamic circulation, characterized by elevated cardiac output, low systemic vascular resistance, and increased plasma volume. Portal hypertension drives splanchnic vasodilation through excessive nitric oxide (NO) production, leading to arterial underfilling and compensatory activation of the renin–angiotensin–aldosterone system (RAAS), sympathetic nervous system, and vasopressin pathways [37]. Initially adaptive, this circulatory state becomes maladaptive, leading to chronic myocardial strain, left ventricular hypertrophy, impaired diastolic function, and progressive ventricular remodeling. Oxidative-stress-mediated endothelial injury and profound mitochondrial dysfunction appear to play a major mechanistic role in these hemodynamic disturbances, promoting microvascular dysregulation and organ damage [18,24,38].

### 2.2. Inflammatory Pathways and Cytokine-Mediated Cardiac Injury

Kupffer cells in the cirrhotic liver release inflammatory cytokines—particularly tumor necrosis factor-alpha (TNF-α), interleukin-6 (IL-6), and transforming growth factor-beta (TGF-β)—which enter systemic circulation and contribute to the systemic inflammatory milieu characteristic of cirrhosis and are implicated in extrahepatic organ dysfunction [39]. These cytokines contribute to adverse cardiac remodeling by promoting cardiomyocyte apoptosis, extracellular matrix remodeling with fibrosis, and impaired excitation–contraction coupling, ultimately leading to systolic dysfunction [40]. In cirrhosis, sustained elevations in inflammatory cytokines such as TNF-α and IL-6 contribute to systemic redox activation and oxidative stress. These cytokines can stimulate NADPH-oxidase-dependent ROS generation in cardiac and vascular cells, thereby amplifying redox-sensitive signaling pathways. Importantly, studies in human myocardium demonstrate increased NOX2 expression and activity in pathological conditions, linking NADPH-oxidase-derived ROS with myocardial injury and adverse remodeling [41,42,43]. Consistently, NOX2 is a major myocardial source of ROS during the progression from hypertrophy to heart failure [44]. TGF-β-related immune and signaling pathways are strongly implicated in pulmonary arterial remodeling and disease progression in pulmonary arterial hypertension [45].

### 2.3. Neurohumoral and Endothelial Dysregulation

Cirrhosis induces a chronic state of neurohumoral activation, involving excessive levels of catecholamines, endothelin-1, vasopressin, and aldosterone. These mediators contribute to systemic vasodilation, water and sodium retention, impaired baroreceptor function, and direct myocardial toxicity. Aldosterone promotes a pro-inflammatory and profibrotic phenotype in the heart through mineralocorticoid-receptor-dependent pathways, which contributes to adverse cardiac remodeling [46]. Endothelin-1 plays a central role in portopulmonary hypertension (PoPH), promoting pulmonary vascular smooth muscle hypertrophy and increased pulmonary vascular resistance (PVR) [47]. Endothelial dysfunction, mediated by oxidative stress, further contributes to pulmonary hypertension [48].

## 3. Oxidative Stress as a Mediator in the Cardiohepatic Axis

Oxidative stress (OS) plays an important role in the crosstalk between hepatic injury and cardiovascular dysfunction. It reflects an imbalance between increased formation of reactive oxygen and nitrogen species and diminished antioxidant defenses, including glutathione, superoxide dismutase, and catalase [49].

### 3.1. Major Sources of Oxidative Stress in Cirrhosis

NADPH oxidase (NOX1/2/4), mitochondrial dysfunction, CYP2E1-dependent metabolism, xanthine oxidase activity, myeloperoxidase (MPO), and ferroptosis represent major, mutually reinforcing contributors to ROS generation. NOX-derived ROS activate hepatic stellate cells, stimulate collagen deposition, and promote cardiomyocyte hypertrophy through redox-sensitive signaling cascades. In parallel, CYP2E1 induction and xanthine oxidase accelerate lipid peroxidation and endothelial injury, particularly in states of alcohol exposure, inflammation, and metabolic stress.

Mitochondrial ROS (mtROS) impair ATP production, disrupt calcium homeostasis, and trigger apoptotic pathways in both hepatic and cardiac tissues, further perpetuating tissue damage. Importantly, these processes seldom occur in isolation; rather, they interact in a feed-forward cycle that amplifies fibrosis, inflammation, and contractile dysfunction [50,51,52,53,54,55,56].

### 3.2. Mitochondrial Dysfunction

Mitochondrial injury in cirrhosis is characterized by loss of membrane potential, accumulation of mtDNA mutations, and opening of the mitochondrial permeability transition pore (mPTP). These events culminate in ATP depletion, impaired oxidative phosphorylation, and activation of intrinsic apoptotic signaling. In hepatocytes, this process accelerates fibrosis progression and promotes susceptibility to ischemia–reperfusion injury.

Within the myocardium, mitochondrial dysfunction compromises energetic reserve and reduces the heart’s ability to respond to physiologic stress. Consequences include impaired calcium reuptake, diminished lusitropy, and progressive diastolic dysfunction—even in the presence of preserved ejection fraction. Over time, persistent mitochondrial failure contributes to structural remodeling and exercise intolerance [57,58].

### 3.3. Nitrosative Stress and Electrophysiological Consequences

Nitric-oxide (NO) dysregulation in cirrhosis exerts complex systemic and cardiac effects. Excessive NO production drives peripheral vasodilation and contributes to the hyperdynamic circulation; however, it also exerts direct negative inotropic influences on cardiomyocytes. Reaction of NO with superoxide generates peroxynitrite (ONOO^−^), which nitrates sarcoplasmic reticulum proteins, damages ion channels, and alters action potential repolarization.

These alterations promote QT prolongation, autonomic imbalance, and heightened arrhythmogenic risk. Thus, nitrosative stress provides a mechanistic bridge linking systemic vasodilation with myocardial depression and electrophysiological instability [26,59].

### 3.4. Ferroptosis in Cardiohepatic Injury

Ferroptosis—an iron-dependent, non-apoptotic form of regulated cell death—is driven by the accumulation of lipid peroxides and depletion of glutathione peroxidase activity. In cirrhosis, excessive intestinal iron absorption, combined with reduced hepcidin synthesis, fosters systemic iron overload, creating a biochemical milieu conducive to ferroptosis.

Within cardiomyocytes, ferroptotic injury promotes lipid peroxidation, mitochondrial swelling, myocardial fibrosis, and progressive impairment of diastolic relaxation. Hepatic ferroptosis further accelerates fibrosis, illustrating how iron-driven oxidative injury simultaneously amplifies both hepatic and cardiac pathology [21,60,61,62,63,64].

### 3.5. Oxidative Stress-Induced Endothelial Dysfunction and Pulmonary Vascular Remodeling

Oxidative stress disrupts endothelial integrity, promotes endothelial-to-mesenchymal transition (EndoMT), stimulates smooth muscle proliferation, and increases extracellular matrix deposition within the pulmonary vasculature. These structural alterations elevate pulmonary vascular resistance (PVR) and impair vasodilatory capacity [65].

As remodeling progresses, increasing afterload imposes chronic pressure stress on the right ventricle, facilitating RV–PA uncoupling and worsening outcomes in portopulmonary hypertension (PoPH). This mechanism highlights the central role of redox imbalance in transitioning from compensated hyperdynamic physiology to clinically significant pulmonary vascular disease [66,67].

### 3.6. Linking Oxidative Stress to Echocardiographic Markers of Subclinical Dysfunction

Oxidative stress-mediated myocardial injury may manifest functionally before conventional echocardiographic indices become abnormal. Advanced strain imaging can detect subtle deformation abnormalities that reflect early mechanical impairment, while RV-focused indices quantify the hemodynamic burden imposed by pulmonary vascular remodeling.

Parameters such as TAPSE, RV free-wall strain, E/e′ ratio, and the TAPSE/PASP ratio provide non-invasive markers of incipient mechanical dysfunction and evolving RV–PA uncoupling. When interpreted alongside biochemical and clinical data, these indices facilitate earlier recognition of cardiomyopathy and support longitudinal monitoring [68].

### 3.7. Clinical Implications of Oxidative Stress in Cardiohepatic Disease

Oxidative stress correlates closely with the Model for End-Stage Liver Disease (MELD) score and functions as an independent predictor of mortality in cirrhosis, underscoring its prognostic relevance. Elevated oxidative biomarkers signal heightened vulnerability to hemodynamic collapse, arrhythmias, and peri-procedural complications.

Integrating redox markers with imaging findings and clinical scoring systems enhances risk stratification in liver transplant candidates, informs decision-making regarding TIPS placement, and refines therapeutic approaches in portopulmonary hypertension. Ultimately, recognizing oxidative stress as a central driver rather than a secondary byproduct reframes cardiohepatic disease as a modifiable, mechanism-based target [69,70,71,72,73]. It should be noted that a substantial portion of these findings derives from experimental and preclinical studies, while clinical data remain limited and heterogeneous.

## 4. Oxidative Stress and Cardiac Remodeling in Cirrhosis

**Figure 2:**
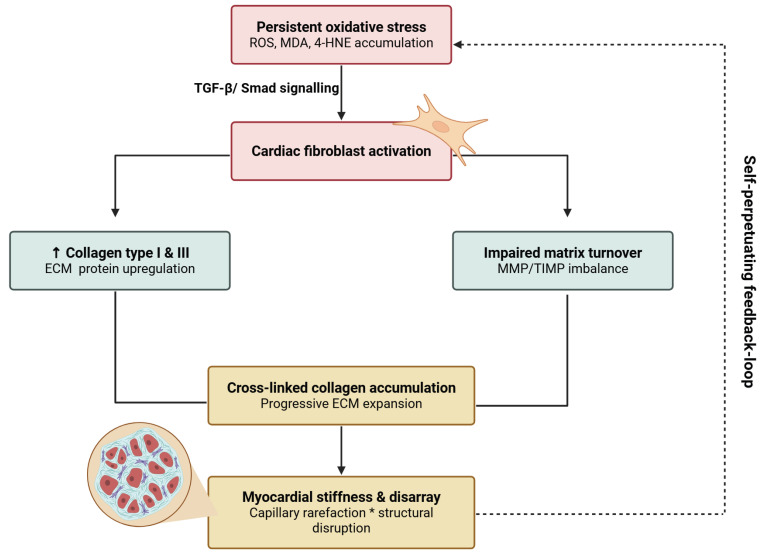
**Oxidative stress-mediated cardiac fibrosis and extracellular matrix remodeling**. Persistent oxidative stress, characterized by the accumulation of reactive oxygen species (ROS) and lipid peroxidation products (e.g., MDA, 4-HNE), activates TGF-β/Smad signaling and drives cardiac fibroblast activation. This leads to increased collagen type I and III synthesis and impaired extracellular matrix (ECM) turnover due to matrix metalloproteinase (MMP)/tissue inhibitor (TIMP) imbalance. The resulting cross-linked collagen accumulation promotes progressive ECM expansion, myocardial stiffening, and microstructural disorganization, including capillary rarefaction. These changes contribute to impaired cardiac function and are sustained by a self-perpetuating redox-dependent feedback loop, reinforcing ongoing fibrotic remodeling. (ROS—reactive oxygen species; MDA—malondialdehyde; 4-HNE—4-hydroxynonenal; ECM—extracellular matrix). Created in BioRender. Blagojević, N. (2026) https://BioRender.com/wnyk1ht, accessed on 6 April 2026.

Oxidative stress represents a pivotal mechanistic link between cirrhosis and the structural, functional, and electrophysiological remodeling of the heart. Unlike classical cardiomyopathies, cirrhotic cardiomyopathy is typically characterized by a preserved left ventricular ejection fraction at rest but an impaired contractile reserve under stress, accompanied by early diastolic dysfunction, subtle abnormalities in myocardial strain, autonomic dysregulation, and clinically relevant QT-interval prolongation. These alterations illustrate how chronic redox and neurohumoral activation can translate into subclinical myocardial dysfunction that becomes clinically manifest under physiological or interventional stress. [74,75,76] Figure 2.

Although cirrhotic cardiomyopathy has traditionally been characterized by left ventricular systolic and diastolic dysfunction, increasing evidence suggests that right ventricular involvement may also occur, particularly in advanced cirrhosis and during stress conditions such as acute decompensation or liver transplantation. However, current data do not clearly demonstrate chamber-specific differences in oxidative stress between the left and right ventricles. Most mechanistic evidence relates to global myocardial or predominantly left-sided dysfunction, while the role of redox signaling in right ventricular impairment remains insufficiently characterized and represents an important area for future research.

### 4.1. Structural Remodeling: Cardiac Fibrosis and Extracellular Matrix Expansion

Persistent oxidative stress activates cardiac fibroblasts through TGF-β/Smad signaling, upregulating collagen types I and III and promoting progressive expansion of the extracellular matrix. Accumulation of cross-linked collagen stiffens the myocardium and disrupts normal architecture. Lipid peroxidation products such as malondialdehyde (MDA) and 4-hydroxynonenal (4-HNE), which accumulate under conditions of oxidative stress, have been implicated in various oxidative injury pathways that influence cellular signaling and protein function. Although direct evidence linking these aldehydes to endothelial-to-mesenchymal transition and activation of cardiac fibroblasts is limited, they reflect the burden of lipid peroxidation that accompanies fibrotic remodeling. In parallel, capillary rarefaction and impaired matrix turnover establish a self-perpetuating loop in which fibrosis feeds back to maintain oxidative stress and structural disarray [77,78,79].

### 4.2. Diastolic Dysfunction and Impaired Relaxation

Diastolic dysfunction often represents the earliest functional manifestation of cirrhotic cardiomyopathy. Oxidative modification of titin, impaired calcium reuptake, and diffuse interstitial fibrosis collectively increase ventricular stiffness and blunt relaxation, even in the presence of preserved systolic ejection fraction. These abnormalities limit the heart’s ability to augment filling during stress, predisposing to pulmonary congestion and exercise intolerance. Peroxynitrite-mediated nitration of sarcomeric proteins further exacerbates lusitropic failure, translating molecular oxidative injury into hemodynamically measurable diastolic impairment. Over time, these subclinical alterations evolve from reversible relaxation abnormalities to more fixed patterns of restrictive filling [59,80,81,82].

### 4.3. Right Ventricular Dysfunction and Advanced Echocardiographic Assessment in Cirrhosis

Right ventricular (RV) dysfunction is increasingly recognized as a clinically relevant complication of cirrhosis, particularly in the setting of portopulmonary hypertension. Chronic oxidative and inflammatory stress promote pulmonary vascular remodeling and progressive elevation in pulmonary vascular resistance, imposing a sustained pressure overload on the right ventricle. In the early phase, compensatory hypertrophy helps maintain stroke volume; however, persistent afterload ultimately results in RV dilation, reduced contractility, functional tricuspid regurgitation, and rising right-sided filling pressures [83,84].

Assessment of RV–pulmonary artery (RV–PA) coupling using the ratio of tricuspid annular plane systolic excursion to pulmonary arterial systolic pressure (TAPSE/PASP) provides a practical, non-invasive index of RV contractile reserve. Lower values indicate emerging uncoupling between RV performance and afterload and have been consistently associated with adverse prognosis. Thresholds around ~0.30–0.32 mm/mmHg have been proposed as markers of impaired RV–PA coupling and early RV dysfunction, often preceding overt right-sided failure [74].

Beyond conventional indices, advanced echocardiographic techniques provide incremental insight into RV mechanics. Speckle-tracking-derived RV free-wall strain detects subtle deformation abnormalities and can reveal early myocardial impairment before TAPSE or fractional area change decline. Because strain analysis is less dependent on loading conditions, it more closely reflects intrinsic myocardial performance [85,86,87].

In cirrhosis, abnormalities in RV strain and other functional indices are frequently observed, even in the absence of overt pulmonary hypertension, underscoring the vulnerability of the right ventricle in this setting. When incorporated into routine surveillance, RV strain and RV–PA coupling indices complement each other, enhance risk stratification, and may help track therapeutic responses over time [88].

### 4.4. Electrophysiological Abnormalities and QT Prolongation

QT-interval prolongation is a characteristic electrophysiological feature of cirrhosis and has been reported in approximately one-third to one-half of patients with advanced disease. Experimental and clinical studies suggest that alterations in nitric oxide-related signaling and potassium channel function contribute to delayed ventricular repolarization, thereby increasing susceptibility to arrhythmias. Clinically, prolonged QTc carries prognostic significance, and values exceeding commonly accepted sex-specific thresholds warrant careful evaluation. Importantly, QT prolongation may fluctuate in parallel with changes in portal pressure, systemic inflammation, and electrolyte balance, underscoring the need for dynamic monitoring rather than single-time-point interpretation [89,90,91,92].

### 4.5. Biomarkers of Oxidative and Cardiac Injury

A panel-based biomarker approach can capture complementary dimensions of redox imbalance, myocardial stress, and overt cardiac injury in cirrhosis. Among the most widely studied oxidative stress markers in chronic liver disease are malondialdehyde (MDA) and F_2_-isoprostanes (e.g., 8-epi-prostaglandin F_2_α), which reflect lipid peroxidation and have been shown to increase with cirrhosis severity and hepatic decompensation [70]. In parallel, systemic indices such as the reduced/oxidized glutathione ratio (GSH/GSSG) and related antioxidant enzymes (superoxide dismutase, catalase, glutathione peroxidase) provide integrated information on the capacity to buffer reactive oxygen and nitrogen species [93].

Beyond generic redox markers, more specific enzymatic sources of oxidative stress can be interrogated. Activation of NADPH oxidase isoforms, particularly NOX2, has been linked to heightened oxidative burden and adverse outcomes across a spectrum of cardiovascular diseases, including heart failure and atrial fibrillation, and is associated with increased formation of F_2_-isoprostanes [94]. Circulating NOX2-derived peptides and F_2_-isoprostanes may therefore serve as mechanistically grounded indicators of ongoing ROS generation in patients with advanced cirrhosis and cardiohepatic involvement, although their incremental prognostic value over conventional risk markers in this population still requires validation [69].

Markers of inflammatory activation and myocardial remodeling further complement the redox profile. Myeloperoxidase (MPO) has been associated with adverse clinical outcomes in heart failure, reflecting leukocyte-driven oxidative stress and endothelial injury, while soluble ST2 (sST2) integrates information on myocardial wall stress and profibrotic signaling [95]. Elevated sST2 levels consistently predict mortality and rehospitalization in both acute and chronic heart failure and provide prognostic information that is additive to, and in some settings partially independent of, natriuretic peptides. Recent data specifically in cirrhotic cardiomyopathy suggest that sST2, in combination with natriuretic peptides, reflects subclinical myocardial remodeling and diastolic dysfunction, thereby supporting its potential role in refined risk stratification in this setting [96].

Natriuretic peptides and cardiac troponins remain central biomarkers at the interface of liver and heart disease. Multiple studies have demonstrated that BNP and NT-proBNP levels correlate with cirrhosis severity, echocardiographic indices of systolic and diastolic dysfunction, and medium-term mortality, supporting their use as screening and prognostic tools in cirrhotic cardiomyopathy [97]. In particular, elevated NT-proBNP in patients with cirrhosis on the liver transplant waiting list has been associated with a higher risk of developing acute-on-chronic liver failure and increased waitlist mortality [98]. High-sensitivity cardiac troponin elevations are less specific in advanced liver disease but may signal concomitant myocardial injury or demand ischemia and have been linked to worse outcomes in decompensated states in broader heart failure cohorts [99].

Taken together, these data support a multimarker strategy in advanced cirrhosis and cardiohepatic disease, in which oxidative stress indices (MDA, F_2_-isoprostanes, NOX2 activity, GSH/GSSG), inflammatory and remodeling markers (MPO, sST2), and cardiac stress/injury markers (NT-proBNP, BNP, high-sensitivity troponin) are interpreted in an integrated fashion. Rather than replacing natriuretic peptides, redox- and remodeling-oriented biomarkers appear to offer complementary information, sometimes identifying heightened risk even when NT-proBNP elevations are modest, and may help distinguish transient hemodynamic congestion from more advanced structural remodeling when measured serially over time [69] Table 1.

### 4.6. Integrative View: Aligning Imaging and Biomarkers in Cardiohepatic Disease

An integrated evaluation that combines echocardiographic indices with biomarkers of oxidative and myocardial stress may offer a comprehensive, mechanistically anchored framework for risk assessment in cirrhosis. Biomarkers reflecting redox imbalance and profibrotic activation could help identify patients with subclinical myocardial injury, while imaging-derived parameters such as TAPSE/PASP and right ventricular free-wall strain quantify the functional consequences of this injury on RV–pulmonary artery coupling and global performance.

This combined approach could enhance diagnostic precision and facilitate more individualized clinical decisions—including selection of patients who may benefit from earlier therapeutic escalation, intensified surveillance, or closer peri-procedural management around high-risk interventions such as TIPS or liver transplantation.

From a pathophysiological standpoint, oxidative stress emerges as one of the pivotal links between liver disease and cardiac remodeling, contributing to extracellular matrix expansion, impaired ventricular compliance, and early diastolic dysfunction. Right ventricular involvement is frequent, particularly in portopulmonary hypertension, where chronic pressure overload promotes progressive RV–PA uncoupling. Advanced echocardiographic techniques, especially RV strain analysis, can reveal these changes before traditional measurements deteriorate and may align with biochemical signals of increased oxidative burden. In parallel, nitrosative injury may contribute to electrical instability and QT-interval prolongation.

Taken together, integrating biomarkers with modern imaging may provide a more nuanced picture of cardiohepatic risk than either modality alone, underscoring the clinical relevance of oxidative-stress-driven remodeling across the spectrum of cirrhosis.

In practical terms, this integrative approach may be conceptualized as a stepwise clinical framework: initial risk stratification based on liver disease severity (e.g., MELD or Child–Pugh score), followed by parallel assessment of oxidative stress and myocardial injury biomarkers, and subsequent refinement using advanced echocardiographic parameters—particularly those reflecting right ventricular–pulmonary arterial coupling. Patients exhibiting concordant abnormalities across these domains could be classified as a high-risk cardiohepatic phenotype, potentially warranting closer monitoring, earlier therapeutic optimization, and careful peri-interventional planning.

Conversely, discordant findings may identify transitional or subclinical stages of disease, where longitudinal reassessment becomes particularly relevant. Although this framework requires prospective validation, it provides a clinically oriented model that may facilitate the translation of mechanistic insights into individualized patient management strategies.

## 5. Therapeutic Strategies Targeting Redox Imbalance in the Cardiohepatic Syndrome

Given that oxidative stress (OS) is a driver of both hepatic fibrogenesis and cardiac remodeling, therapeutic strategies targeting redox imbalance have attracted increasing attention. Contemporary approaches encompass conventional antioxidants, agents with indirect antioxidant properties, mitochondria-targeted therapies, NOX inhibition, ferroptosis blockade, and emerging regenerative or precision-guided interventions Table 2. Most of the therapeutic approaches discussed remain supported primarily by preclinical or early-phase clinical data, with limited high-quality evidence from large clinical trials.

### 5.1. Conventional Antioxidants

N-acetylcysteine (NAC) replenishes intracellular glutathione (GSH) pools and directly scavenges reactive oxygen species, thereby restoring redox balance during periods of heightened oxidative demand. Beyond its established role in acetaminophen toxicity, NAC has been shown to improve systemic and hepatic perfusion, modulate inflammatory signaling, and protect hepatocytes under ischemia–reperfusion stress.

Vitamin E attenuates lipid peroxidation and stabilizes cell membranes, with downstream effects on mitochondrial integrity and endothelial function.

Phytotherapeutic compounds, such as silymarin and curcumin, exert pleiotropic antioxidant and anti-inflammatory actions. By modulating NF-κB signaling, inhibiting TGF-β-driven profibrotic pathways, and enhancing endogenous antioxidant defenses, these agents may mitigate hepatic—and potentially extrahepatic—fibrotic remodeling. Although most data derive from preclinical or small clinical studies, the collective evidence supports their role as adjunctive, mechanism-targeted therapies within a broader cardiohepatic framework [100,101,102,103].

### 5.2. Drugs with Indirect Antioxidant Properties

Several widely used cardiorenal and hepatoprotective agents exert clinically meaningful redox-modulating effects despite not being classified as conventional antioxidants. Spironolactone, through mineralocorticoid receptor blockade, reduces myocardial fibrosis and ventricular stiffness by suppressing TGF-β-mediated signaling and attenuating oxidative injury, effects that contribute to its established prognostic benefit in advanced heart failure.

Statins, beyond lipid lowering, suppress NADPH oxidase activity, improve endothelial nitric oxide bioavailability, and exert favorable effects on intrahepatic vascular resistance, resulting in reduced portal pressure in patients with cirrhosis and portal hypertension. Experimental and limited clinical data further suggest that these pleiotropic vascular and anti-inflammatory effects may indirectly influence right ventricular remodeling by reducing afterload in conditions of pulmonary vascular dysfunction.

Similarly, ACE inhibitors and angiotensin receptor blockers blunt angiotensin II-driven reactive oxygen species generation, thereby attenuating downstream inflammatory and profibrotic remodeling. These mechanisms, originally characterized in renal disease, represent shared redox-sensitive pathways relevant to maladaptive remodeling across the heart and liver [104,105,106]. Collectively, such pleiotropic actions help explain the prognostic benefit of neurohormonal blockade in advanced cardiovascular disease and suggest potential therapeutic relevance within the complex hemodynamic milieu of cirrhosis, particularly when congestion, neurohormonal activation, and oxidative stress coexist.

### 5.3. Mitochondria-Targeted Antioxidant Therapies

MitoQ, coenzyme Q10, melatonin, and elamipretide selectively localize to mitochondria, stabilize inner membrane structure, and decrease mitochondrial reactive oxygen species (mtROS), thereby preserving ATP generation and limiting apoptosis. By improving bioenergetic efficiency and targeting oxidative injury at its intracellular source, these agents address a central node in cardiohepatic pathophysiology.

Experimental models demonstrate that MitoQ ameliorates hypoxia-induced pulmonary hypertension and limits right ventricular hypertrophy, supporting mitochondria as a promising therapeutic target in cardiopulmonary disease. Elamipretide, through its interaction with cardiolipin, improves mitochondrial coupling and bioenergetic efficiency in the failing human heart and may enhance myocardial performance under conditions of stress.

Collectively, mitochondria-directed therapies represent an evolving class of interventions aimed at restoring cellular energetics rather than merely counteracting downstream oxidative damage [107,108].

### 5.4. NOX Inhibitors and Redox Signaling Modulators

Targeting NADPH oxidase (NOX) is a rational strategy to blunt oxidative stress at its origin, given that NOX enzymes represent a major source of ROS in hepatic stellate cells and play a central role in experimental liver fibrogenesis. In preclinical models, apocynin attenuates liver fibrosis events while modulating oxidative stress, inflammatory mediators, and apoptosis. Likewise, the NOX1/4 inhibitor GKT137831 suppresses stellate cell activation and reduces experimental liver fibrosis, supporting NOX-driven ROS as an upstream, disease-modifying pathway. Importantly, NOX inhibition impacts multiple downstream cascades—including profibrotic TGF-β signaling and inflammatory recruitment—highlighting its potential to modify core fibrogenic biology rather than merely neutralize ROS byproducts [109,110].

### 5.5. Ferroptosis Inhibitors

Ferroptosis is an iron-dependent, lipid peroxidation-driven form of regulated cell death defined by glutathione depletion and functional inactivation of glutathione peroxidase 4 (GPX4), which normally protects membrane phospholipids from oxidative damage. Genetic or functional loss of GPX4 results in unchecked lipid peroxidation and rapid organ failure in vivo, establishing ferroptosis as a fundamental redox-dependent mechanism of tissue injury [111].

Growing preclinical evidence implicates ferroptosis in the progression of chronic liver and cardiovascular diseases. In experimental models, iron overload and impaired antioxidant defenses promote lipid peroxidation within hepatocytes and hepatic stellate cells, accelerating fibrogenic activation and extracellular matrix deposition. Similarly, cardiomyocytes exhibit heightened susceptibility to ferroptotic injury under conditions of metabolic stress, ischemia, and inflammation, linking iron-dependent lipid toxicity to myocardial cell loss and adverse remodeling. These observations position ferroptosis at the intersection of oxidative stress, metabolic dysregulation, and tissue fibrosis across the cardiohepatic axis.

Pharmacologic modulation of ferroptosis has therefore emerged as a potential therapeutic strategy. Iron chelators such as deferoxamine reduce the labile iron pool and limit lipid peroxidation, while specific ferroptosis inhibitors, including liproxstatin-1, directly suppress lipid radical propagation and preserve cellular viability in multiple experimental models. Beyond preventing cell death, ferroptosis inhibition has been shown to attenuate fibrotic remodeling and microvascular injury in preclinical settings, suggesting disease-modifying effects rather than purely cytoprotective action.

Although clinical translation remains in its infancy, targeting ferroptosis introduces a novel therapeutic dimension focused on preserving cellular integrity and bioenergetic competence in environments dominated by oxidative and metabolic stress. Within the context of cirrhosis-associated cardiomyopathy and advanced cardiohepatic disease, ferroptosis represents a mechanistically attractive target linking iron dysregulation, redox imbalance, and progressive organ dysfunction [20,21,61,111].

### 5.6. Biomarker-Guided Therapeutic Perspectives

Redox-related biomarkers—including malondialdehyde (MDA), indices of NOX2 activity, soluble ST2, and N-terminal pro-B-type natriuretic peptide (NT-proBNP)—provide complementary information linking oxidative burden with myocardial stress and remodeling. Experimental and clinical data support the role of oxidative stress markers in reflecting systemic redox imbalance, while cardiac stress biomarkers such as sST2 and NT-proBNP offer validated prognostic value in heart failure.

The integrated interpretation of these biomarkers may enhance risk stratification by distinguishing stable from evolving myocardial dysfunction and identifying patients at increased risk of decompensation. In advanced liver disease, biomarker trajectories may complement imaging and hemodynamic assessment by capturing dynamic biological stress that is not fully reflected by structural measures alone. Moreover, in early-phase studies of redox-modulating therapies, such markers represent attractive exploratory endpoints capable of detecting biological response prior to overt clinical change [112,113,114].

### 5.7. Role in Transplant Assessment

Both latent cardiac dysfunction and limited cardiopulmonary reserve critically influence outcomes after TIPS placement or liver transplantation, predisposing vulnerable patients to acute right ventricular failure, arrhythmias, and hemodynamic instability. Conventional preoperative assessment may underestimate this risk when it relies predominantly on resting systolic indices.

Contemporary evaluation therefore emphasizes a more comprehensive assessment of right ventricular function and cardiopulmonary reserve, incorporating parameters such as pulmonary artery pressures, indices of right ventricular–pulmonary arterial coupling (e.g., the TAPSE/PASP ratio), and functional testing in selected patients. Advanced echocardiographic techniques and emerging biomarkers may provide additional insight into subclinical vulnerability, although their role in routine transplant pathways continues to evolve. Integrating these approaches has the potential to enhance peri-procedural risk stratification and inform optimization strategies prior to intervention [115].

### 5.8. Personalized Medicine in Cirrhotic Cardiomyopathy: A Redox–Echocardiography Model

Cirrhotic cardiomyopathy is increasingly recognized as a dynamic and progressive condition, characterized by latent myocardial dysfunction that may remain clinically silent under resting conditions but becomes unmasked during physiological stress. Conventional assessments often fail to capture this early vulnerability, as systolic function at rest is frequently preserved [116].

Building on established pathophysiological concepts, the integration of advanced echocardiographic parameters with markers of oxidative stress may offer a conceptual framework for stage-oriented risk assessment in cirrhotic cardiomyopathy. Early disease may be characterized by subclinical myocardial involvement without overt functional impairment, whereas progression is associated with emerging abnormalities in ventricular mechanics, impaired cardiopulmonary coupling, and rising cardiac stress biomarkers. In advanced stages, structural remodeling and electrophysiological disturbances, including QT-interval prolongation, reflect severe myocardial involvement with adverse prognostic significance.

While this integrated, biomarker-informed approach extends beyond current diagnostic standards, it aligns with the evolving understanding of cirrhotic cardiomyopathy as a stress-sensitive disorder and may support dynamic risk surveillance and individualized timing of therapeutic or procedural interventions.

### 5.9. Emerging Therapies Under Investigation

Novel therapeutic strategies—including microRNA modulation, pharmacologic activation of nuclear factor erythroid 2-related factor 2 (Nrf2), and glucagon-like peptide-1 receptor agonists—are under active investigation and may broaden future treatment paradigms in diseases driven by oxidative stress and inflammation. MicroRNA-based approaches offer the potential to simultaneously influence multiple profibrotic and redox-sensitive pathways, particularly in the regulation of hepatic stellate cell activation and liver fibrogenesis.

Pharmacologic activation of Nrf2, a master transcriptional regulator of endogenous antioxidant defenses, represents another promising strategy for counteracting chronic oxidative injury and mitochondrial dysfunction across a range of chronic diseases. Likewise, GLP-1 receptor agonists, originally developed for metabolic disorders, have demonstrated cardiovascular protective effects that extend beyond glucose lowering and include improvements in endothelial function and oxidative stress signaling.

In parallel, stem-cell-based approaches and extracellular vesicle platforms are being explored in preclinical settings as potential means of modulating inflammation, microvascular dysfunction, and tissue repair through paracrine mechanisms. Collectively, these emerging strategies illustrate a conceptual shift from purely symptomatic management toward mechanism-oriented, precision approaches aimed at interrupting redox inflammatory pathways that contribute to progressive organ dysfunction [117,118,119].

Taurine and spermidine have recently emerged as promising metabolic modulators relevant to the cardiohepatic axis. Taurine, a sulfur-containing amino acid with antioxidant and anti-inflammatory properties, contributes to mitochondrial stabilization, regulation of intracellular calcium homeostasis, and attenuation of reactive oxygen species (ROS) generation, thereby protecting both cardiomyocytes and hepatocytes from oxidative injury [120,121]. Spermidine, a naturally occurring polyamine, exerts cardiometabolic protective effects primarily through activation of autophagy, improvement in mitochondrial quality control, and reduction in oxidative stress [122]. Epidemiological and experimental data further suggest that higher spermidine availability is associated with improved cardiovascular outcomes and attenuation of age-related cardiac remodeling [123]. Collectively, these findings highlight taurine and spermidine as potential therapeutic targets for restoring redox balance within the integrated cardiohepatic axis.

## 6. Emerging Role of Cardiometabolic Therapies in the Cardiohepatic Syndrome

Beyond conventional approaches to heart failure and liver disease, cardiometabolic agents—particularly sodium–glucose cotransporter-2 inhibitors (SGLT2i) and glucagon-like peptide-1 receptor agonists (GLP-1 RA)—have drawn increasing attention because of their pleiotropic effects across metabolic, vascular, and cardiac pathways. Although originally developed as glucose-lowering therapies, accumulating clinical and experimental evidence indicates that these agents exert cardiovascular benefits extending beyond glycemic control, including modulation of oxidative stress, improvement in myocardial energetics and mitochondrial efficiency, and attenuation of systemic inflammation.

Through these mechanisms, SGLT2i and GLP-1 RA may indirectly influence pathophysiological processes relevant to the cardiohepatic axis, particularly in settings characterized by metabolic dysfunction, chronic inflammation, and heart failure. Emerging data further suggest potential effects on pulmonary hemodynamics and right ventricular load, although these aspects remain under active investigation. Collectively, these observations position cardiometabolic therapies as promising adjuncts within an integrated, mechanism-oriented approach to advanced cardiovascular disease [124,125,126,127].

### 6.1. SGLT2 Inhibitors

Large outcome trials demonstrate that SGLT2 inhibitors consistently reduce heart failure hospitalizations and improve composite cardiovascular outcomes (including cardiovascular death and HF events), with benefits observed also in patients without diabetes, underscoring class effects that extend beyond glycemic control [128,129,130].

Mechanistically, SGLT2i are thought to attenuate myocardial oxidative stress and improve cellular energetics, with proposed contributions from reduced NADPH oxidase–linked signaling, shifts toward more oxygen-efficient substrate utilization (including ketone metabolism), and potential off-target effects on ionic homeostasis; however, direct inhibition of myocardial NHE-1 remains debated, and data are mixed [131,132].

From the hepatic standpoint, randomized and pooled clinical data in NAFLD/T2D indicate that SGLT2i reduce liver fat and may improve steatosis and fibrosis surrogates, effects largely mediated by improved insulin resistance and systemic inflammatory tone [133,134].

Evidence in established cirrhosis is still limited and mostly derived from small reports and literature reviews; therefore, concerns about volume depletion, prerenal azotemia, and infection risk warrant careful patient selection, close monitoring of kidney function, and avoidance during active sepsis or severe hypotension until prospective trials clarify efficacy and safety in this population [135].

### 6.2. GLP-1 Receptor Agonists

Glucagon-like peptide-1 receptor agonists (GLP-1 RA) reduce major adverse cardiovascular events or demonstrate cardiovascular benefit in patients with type 2 diabetes, while producing clinically meaningful weight loss and improving insulin sensitivity [136,137]. In selected metabolic populations, GLP-1 RA have also been shown to reduce liver fat content and improve histologic features of steatohepatitis [138].

At the molecular level, GLP-1 signaling suppresses oxidative and nitrosative stress, enhances endothelial nitric oxide bioavailability, and down regulates inflammatory and profibrotic pathways, mechanisms that may contribute to cardioprotection and potentially modulate the progression of hepatic fibrogenesis [139]. Emerging experimental and translational data further suggest that GLP-1 RA may reduce epicardial adipose tissue inflammation and improve microvascular function, processes implicated in myocardial dysfunction and arrhythmogenic susceptibility, although direct evidence in cirrhosis remains limited.

Despite these promising effects, gastrointestinal intolerance, risk of volume depletion, and the paucity of data in decompensated cirrhosis necessitate individualized clinical decision-making. Severe cachexia, advanced malnutrition, or pronounced gastroparesis warrant particular caution when considering GLP-1 RA therapy in advanced liver disease.

### 6.3. Conceptual Integration and Research Priorities

Taken together, SGLT2 inhibitors and GLP-1 receptor agonists emerge as promising cardiometabolic therapies capable of modulating systemic inflammation and oxidative stress—pathophysiological processes that link metabolic liver disease with cardiac dysfunction. Robust heart failure outcome data for SGLT2i and mechanistic insights into cardiometabolic crosstalk support the rationale for exploring these agents within the broader cardiohepatic context [140].

Important research priorities include defining safety and efficacy across compensated versus decompensated liver disease, clarifying potential effects on right ventricular function and cardiopulmonary reserve, and determining whether redox-sensitive biomarkers (e.g., indices of oxidative stress and inflammation) can help identify patients most likely to benefit. Additional questions relate to interactions with established heart failure therapies and implications for advanced interventions, including transplant evaluation.

Ultimately, rigorously designed prospective studies specifically enrolling patients with advanced liver disease are required before routine implementation of SGLT2i or GLP-1 RA can be recommended for cirrhosis-associated cardiac dysfunction [140].

## 7. Future Directions and Knowledge Gaps

A unified and clinically applicable definition of cirrhotic cardiomyopathy that incorporates redox-sensitive biomarkers and right-sided cardiac function remains lacking. Current diagnostic frameworks predominantly emphasize left ventricular systolic and diastolic indices, while failing to capture subclinical oxidative injury, right ventricular–pulmonary artery uncoupling, and electrophysiological vulnerability. As a result, many patients remain clinically silent until physiological stress unmasks advanced decompensation. Right ventricular dysfunction, in particular, appears systematically under-recognized, despite its strong association with transplant outcomes, TIPS-related hemodynamic instability, and mortality.

Equally important are the substantial gaps in interventional evidence. Targeted studies addressing ferroptosis, NOX-driven oxidative injury, mitochondrial bioenergetics, or redox-guided therapeutic selection are scarce, with most available data derived from small cohorts or preclinical models. Disease heterogeneity—including compensated versus decompensated cirrhosis and alcohol- versus metabolic-associated etiologies—combined with inconsistent endpoints, further limits translation into routine practice. Pediatric populations, sex-specific differences, and longitudinal trajectories across disease stages remain largely unexplored.

Future research should prioritize the validation of integrated biomarker–imaging algorithms capable of detecting early myocardial injury before overt dysfunction develops. In this context, mitochondria-centered biology represents a particularly compelling avenue, given its central role in oxidative stress, energy failure, and myocardial remodeling. Rigorous evaluation of mitochondria-directed and redox-modulating therapies in adequately powered, prospective studies with clinically meaningful outcomes is urgently needed. Harmonized trial designs, standardized echocardiographic protocols—including right ventricular metrics—and serial assessment of oxidative biomarkers will be essential to establish causality and to define which patients are most likely to benefit from targeted cardiohepatic interventions [141,142,143,144].

Emerging omics technologies, including metabolomics, proteomics, transcriptomics, and microbiome profiling, offer powerful tools for elucidating molecular pathways linking hepatic dysfunction, cardiovascular remodeling, and systemic oxidative stress within the cardiohepatic axis [145,146]. In parallel, artificial intelligence and machine learning approaches enable integration of complex multidimensional datasets, including clinical variables, imaging parameters, and biomarker profiles, thereby improving risk stratification and supporting personalized management strategies [147]. The combination of omics-based profiling with advanced computational analytics may therefore provide deeper insight into the molecular architecture of the cardiohepatic axis and facilitate earlier detection of cardiohepatic interactions [148].

## 8. Conclusions

Oxidative stress represents an important mechanistic bridge linking hepatic fibrosis to cardiac remodeling, integrating mitochondrial dysfunction, NADPH oxidase activation, ferroptosis, endothelial injury, and cytokine-mediated inflammation into a unified pathobiological continuum. Through these interrelated pathways, chronic liver disease progressively shapes a vulnerable cardiac phenotype characterized by extracellular matrix expansion, myocardial stiffness, impaired relaxation, and heightened susceptibility to electrophysiological instability. As remodeling advances, deterioration in right ventricular mechanics and progressive right ventricular–pulmonary artery uncoupling emerge as critical hemodynamic hallmarks that predispose to clinical heart failure and adverse outcomes.

The expanding availability of advanced echocardiographic tools—including myocardial strain imaging and indices of RV–pulmonary artery coupling—together with redox-related biomarkers such as NOX2, malondialdehyde, soluble ST2, and 8-isoprostanes, offers an opportunity for earlier detection and longitudinal surveillance of cirrhosis-associated cardiac dysfunction. Integrating these modalities into a structured diagnostic framework may enable more precise phenotyping, improved risk stratification, and more informed timing of therapeutic or procedural interventions across the natural history of cirrhosis.

Looking forward, targeted strategies aimed at modulating oxidative and mitochondrial pathways—including NOX inhibition, mitochondria-directed antioxidants, ferroptosis modulation, and selected cardiometabolic agents—hold promise for reshaping clinical management. By shifting the paradigm from late recognition of overt cardiomyopathy toward proactive, mechanism-guided prevention and individualized therapy, future cardiohepatic strategies may improve quality of life, procedural safety, and long-term outcomes for patients with advanced liver disease. This perspective supports a transition toward earlier, mechanism-informed evaluation and individualized management of cardiac dysfunction in advanced liver disease.

## Figures and Tables

**Table 1:** Biomarkers of oxidative and cardiac injury.

Category	Biomarker	Pathophysiological Relevance	Translational Status
**Oxidative stress markers**	Malondialdehyde (MDA)	Product of lipid peroxidation reflecting systemic oxidative stress; increases with cirrhosis severity and hepatic decompensation	Experimental/research use
	F_2_-isoprostanes (e.g., 8-epi-PGF_2_α)	Reliable markers of lipid peroxidation and ROS-mediated cellular injury	Experimental/research use
	GSH/GSSG ratio	Indicator of systemic redox balance, reflecting antioxidant buffering capacity	Experimental/research use
	Antioxidant enzymes (SOD, catalase, glutathione peroxidase)	Reflect endogenous antioxidant defense against reactive oxygen and nitrogen species	Experimental/research use
**Sources of ROS generation**	NADPH oxidase activity (NOX2)	Enzymatic source of ROS implicated in cardiovascular remodeling and systemic oxidative burden	Investigational biomarker
	NOX2-derived peptides	Circulating indicators of NADPH oxidase activation and ongoing ROS generation	Investigational biomarker
**Inflammatory and remodeling biomarkers**	Myeloperoxidase (MPO)	Marker of leukocyte-driven oxidative stress, endothelial injury, and inflammatory activation	Investigational biomarker
	Soluble ST2 (sST2)	Reflects myocardial wall stress and profibrotic signaling; a strong prognostic marker in heart failure	Emerging clinical biomarker
**Cardiac stress and injury biomarkers**	NT-proBNP	Marker of myocardial wall stress and volume overload; correlates with cirrhosis severity and cardiac dysfunction	Established clinical biomarker
	BNP	Natriuretic peptide reflecting cardiac stress and neurohormonal activation	Established clinical biomarker
	High-sensitivity cardiac troponin	Marker of myocardial injury that may indicate concomitant cardiac damage in advanced cirrhosis	Established clinical biomarker

**Table 2:** Therapeutic strategies targeting redox imbalance in the cardiohepatic axis.

Therapy Class	Representative Agents	Primary Target(s)	Mechanism of Action	Level of Evidence
**Conventional antioxidants**	N-acetylcysteine (NAC)	GSH depletion, ROS	Replenishes intracellular glutathione, directly scavenges ROS, and improves perfusion and inflammatory balance	Clinical use (established in specific settings) + small clinical studies
	Vitamin E	Lipid peroxidation, membranes	Inhibits lipid peroxidation, stabilizes membranes, and preserves mitochondrial and endothelial function	Small clinical studies/limited evidence
	Silymarin, curcumin	NF-κB, TGF-β pathways	Anti-inflammatory and antifibrotic effects; enhances endogenous antioxidant systems	Preclinical + small clinical studies
**Drugs with indirect antioxidant effects**	Spironolactone	MR signaling, TGF-β	Reduces fibrosis, suppresses oxidative injury, and improves ventricular remodeling	Strong clinical evidence (CV disease)
	Statins	NOX activity, endothelial NO	Reduces ROS production, improves endothelial function, and decreases portal pressure	Clinical + translational evidence
	ACE inhibitors/ARBs	Ang II–ROS axis	Reduces Ang II-mediated ROS generation and downstream fibrosis	Strong clinical evidence (CV disease)
**Mitochondria-targeted therapies**	MitoQ	mtROS	Reduces mitochondrial RO and improves bioenergetics	Preclinical/early clinical
	Coenzyme Q10	Electron transport chain	Enhances mitochondrial function and reduces oxidative stress	Small clinical studies
	Melatonin	mtROS, mitochondrial signaling	Antioxidant + mitochondrial protection	Preclinical/small clinical
	Elamipretide	Cardiolipin, mitochondrial coupling	Improves mitochondrial structure and ATP production	Early-phase clinical
**NOX inhibitors**	Apocynin	NOX enzymes	Reduces ROS production, inflammation, and apoptosis	Preclinical
	GKT137831 (NOX1/4 inhibitor)	NOX1/4, stellate cells	Inhibits fibrogenesis and suppresses TGF-β signaling	Preclinical/early clinical
**Ferroptosis inhibitors**	Deferoxamine	Iron overload	Reduces labile iron pool and lipid peroxidation	Preclinical/limited clinical
	Liproxstatin-1	Lipid peroxidation chain	Inhibits ferroptosis and preserves cellular viability	Preclinical
**Metabolic modulators of redox balance**	Taurine	Mitochondrial stability, intracellular Ca^2+^ homeostasis, ROS generation	Antioxidant and anti-inflammatory effects; stabilizes mitochondria; regulates calcium homeostasis; attenuates ROS-mediated injury in cardiomyocytes and hepatocytes	Experimental/early translational
	Spermidine	Autophagy pathways, mitochondrial quality control, oxidative stress	Activates autophagy; improves mitochondrial function and quality control; reduces oxidative stress; attenuates age-related cardiac remodeling	Experimental/emerging (epidemiological + preclinical data)

## Data Availability

No new data were created or analyzed in this study. Data sharing is not applicable to this article.
